# Patient characteristics and degrees of discontinuity of care in Danish general practice: a cohort study

**DOI:** 10.3399/BJGP.2024.0570

**Published:** 2025-03-20

**Authors:** Ditte Elschner Rimestad, Peder Ahnfeldt-Mollerup, Troels Kristensen

**Affiliations:** 1 Department of Clinical medicine & Department of Pediatrics, Aarhus University, Aarhus, Denmark; 2 Danish Centre for Health Economics, Department of Public Health, University of Southern Denmark, Odense, Denmark; 3 Research Unit of General Practice, Department of Public Health, University of Southern Denmark, Odense, Denmark

**Keywords:** family medicine, continuity of patient care, demography, socioeconomic factors, healthcare disparities, morbidity, geographic locations

## Abstract

**Background:**

Continuity of care is crucial for effective health care, but patients often experience discontinuity of care. Understanding the patterns and associated patient characteristics can guide interventions to improve continuity of care.

**Aim:**

To investigate longitudinal provider discontinuity of care to identify disparities in patient characteristics across degrees of discontinuity of care in Danish general practice.

**Design & setting:**

Cohort study using data from all Danish general practice patients aged ≥12 years, who were alive between 2007 and 2018.

**Method:**

Logistic regression was employed to estimate discontinuity of care at level 0 versus: 1–2 shifts, 3–4 shifts, 5–6 shifts, 7–8 shifts, and ≥9 shifts. Five regression models were used to analyse the odds of different levels of discontinuity of care relative to demographic, regional, municipal, socioeconomic, and morbidity factors.

**Results:**

The majority of males were likely to have lower levels of discontinuity of care than females; however, males in the subgroup with the most shifts had a higher likelihood of experiencing the highest level of discontinuity. Younger adults aged 25–44 years had a higher likelihood of moderate-to-high discontinuity of care compared to those aged 12–24 years, whereas older adults had a lower probability. Discontinuity of care was higher among residents of the Capital and Zealand Regions, and varied by municipality type — being lowest in intermediate areas and highest in peripheral and rural municipalities at the most severe levels. Individuals who were unemployed, those in the lower income quartiles, and those classified as being of ‘other ethnicities’ and single status had increased probability of discontinuity of care. Patients with lower or moderate-to-high morbidity were also more likely to experience discontinuity of care than patients with no chronic diseases.

**Conclusion:**

This study revealed disparities in discontinuity of care linked to lower socioeconomic status and higher morbidity, with varying odds by region, municipality type, age, and gender.

## Introduction

Continuity of care (COC) is recognised as a cornerstone of effective health care, which ensures that patients receive consistent and coordinated services.^1–4^ Long-term COC is crucial for good patient outcomes, and promotes consistency, understanding, and better chronic disease management.^5^ However, patients encounter discontinuity of care, which can negatively affect health outcomes.^4,6,7^ Discontinuity of care occurs when there are breaks in a patient’s care pathway, leading to fragmented services.^3^ Previous literature has suggested that achieving COC for patients with complex care needs is facilitated by professional and cross-disciplinary cooperation;^8–10^ as such, it is crucial to develop provider COC, ensuring that patients remain under the care of the same clinical team for a longer period.^11,12^

There are two main perspectives on COC in general practice: one focuses on the patient’s experience of a continuous, caring relationship with a health professional; the other emphasises the provider’s role in delivering seamless services through coordination and information sharing among healthcare staff. This study focused on discontinuity of care at the provider level and the degrees of site discontinuity, which refers to the number of changes in registration between general practice providers over an extended analysis period.^3,11^ 1

**How this fits in**

Continuity of care is a well-established component of high-quality general practice, but research on discontinuity remains limited. Most studies focus on personal continuity, use small samples or short follow-up periods, and treat discontinuity simply as the absence of continuity. Although recent work has introduced more nuanced continuity measures and examined patient characteristics, variation in the degree and distribution of discontinuity across patient groups remains underexplored. Few studies have examined discontinuity at the clinic level, particularly in the form of shifts between providers over time. This study addresses that gap by investigating longitudinal clinic-level discontinuity in a population-based setting, with particular focus on variation by individual characteristics. It reveals disparities linked to gender, age, geography, socioeconomic status, and morbidity burden, highlighting the need for targeted interventions to improve continuity of care.

The nature and extent of discontinuity of care across patient factors is not well known.^13^ The literature has suggested that demographic, geographic, socioeconomic, and morbidity factors may be linked to the degree of discontinuity of care,^14–19^ but findings have been inconsistent.^18^ The present study examined whether these links hold in Danish general practice. The authors hypothesised that older patients with ≥1 chronic condition would exhibit a higher degree of COC compared to younger and healthier individuals,^14,20^ and that there would be greater discontinuity of care among patients with lower socioeconomic status and those living in urban municipalities.^19–22^ This study aimed to examine levels of discontinuity of care in general practice and key patient disparities with respect to patient characteristics for the entire Danish population.

## Method

### Setting

Danish GPs operate within a public healthcare system that provides free access to services for all residents. GPs serve as the cornerstone, acting as gatekeepers to secondary care and specialists. Registration with a practice ensures access to care and serves as a structural basis for fostering continuity and long-term relationships between patient and provider. Most GPs are self-employed and work in private practices under contracts with one of five regions. In total, 36% of practices are single doctor, while 64% are multidoctor.^23^ General practices typically include support staff — such as nurses, medical secretaries, and medical students — and collaborate with public workers, such as home nurses, social workers, and midwives.

### Approaches

This cohort study was based on data from the Danish list system, in which everyone is listed at provider level.^12^ The authors used a population dataset on provider COC in Danish general practice, dating from 1 January 2007 until 31 December 2018, to analyse population differences and odds for discontinuity of care. The cohort included all listed patients aged ≥12 years, who were alive during the analysis period.^11^ By utilising population data, this study allowed for the analysis of discontinuity of care with minimal concerns about sampling errors.

The authors compared patient characteristics across differing degrees of individual discontinuity of care (0 versus: 1–2 shifts, 3–4 shifts, 5–6 shifts, 7–8 shifts, and ≥9 shifts) at the end of the analysis period. This approach allowed the average patient characteristics within each level of discontinuity of care to be summarised. Patient characteristics included demographic markers (gender and age), regional markers (region of residence), and municipal markers (peripheral, rural, urban, and intermediate) based on 14 classification indicators.^24^ The indicators are related to population density, infrastructure access, labour market integration, age, education, and demographic development. Municipality markers are defined using a composite rural district index ranging from 1 to 100, based on the indicators.^24^ Municipalities with the highest index scores are classified as peripheral municipalities, while those with the lowest scores are considered urban municipalities. Intermediate and rural municipalities fall between these two extremes, being neither highly urbanised nor strongly peripheral.

Socioeconomic markers (unemployed, short education, other ethnicity, single, children at home, and income) were based on data from Statistics Denmark.^25^ Morbidity was assessed using the Charlson Comorbidity Index (CCI), with diagnoses obtained from the Danish National Patient Register, which contains information on all hospital contacts. Disposable income was used as a key socioeconomic indicator, defined by Statistics Denmark as total income, including earned income, capital income, and transfers, after deducting personal taxes and interest payments. Due to the large sample size, differences between discontinuity of care subpopulations and baseline COC (0 shifts) were described without formal hypothesis testing.^26^

At the end of the cohort period in 2018, logistic regression was used to assess probability across discontinuity of care degrees from 2007–2018.^27,28^ Five independent models were created, each representing a different degree of discontinuity of care, with a dichotomous outcome comparing COC (no shifts) with discontinuity of care degrees. Patient characteristics that were hypothesised to be linked to discontinuity of care were included as dichotomous variables in each model. Age, income, and CCI score were categorised into age bands, income quartiles, and morbidity bands (0, 1–2, 3–4, or ≥5 chronic diseases), respectively. All variables were included simultaneously as substantive covariates. The 12–24-year-old age group and the highest income quartile (both of which are often linked to better health), the category with the lowest CCI score (the healthiest group), South Region, and urban municipalities were selected as references. Logistic regressions were adjusted for patient clustering at clinics using cluster-robust standard errors. These models assessed the odds of each discontinuity of care level relative to COC. Data analysis was conducted using Stata (version 16).

## Results

**Figure 1.**
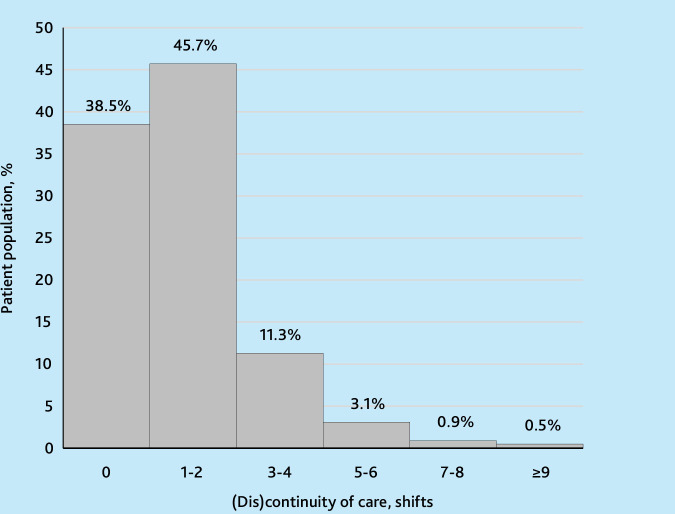
Distribution of continuity of care and varying degree of discontinuity of care among patients in Danish general practice, 2007–2018.

Figure 1 shows the distribution of the population by COC and the degree of discontinuity of care in Danish general practice over the period of analysis. The majority of patients (61.5%) experienced varying degrees of discontinuity of care, with the most common being 1–2 shifts (45.7%) and 3–4 shifts (11.3%). Notably, 38.5% of patients maintained COC over the 12-year period.

Table 1 presents COC and discontinuity of care degrees as a percentage of the total number of patients per variable across the different patient characteristics (horizontally) and as a percentages of the individual shift group (vertically). The proportion of males increased with higher discontinuity: males made up 49.3% of the 0-shift group and 56.5% of the ≥9-shift group. The 25–34-year-old age group had the lowest share of COC (14.1%) of all the age groups, and the highest percentages for 3–4 shifts. From age 45–84 years, the percentage of patients with COC increased with age across each successive age group, but this trend was not seen in the oldest group — those aged ≥85 years.

**Table 1.** Continuity of care and varying degrees of discontinuity of care, by patient characteristic, 2007–2018

Continuity of care and degrees of discontinuity of care, %^a^	*n*	0 shifts	1–2 shifts	3–4 shifts	5–6 shifts	7–8 shifts	≥9 shifts
Total population, *n*	4 571 041	1 758 753	2 089 204	515 175	143 740	43 178	20 991
Mean number of shifts, *n*	4 571 041	0	1.3	3.3	5.4	7.4	10.7
**Demographic**							
Male^b^	2 251 225	49.3	48.8	50.1	52.3	54.1	56.5
Mean age, years^b^	4 571 041	51.9	47.8	38.9	34.1	32.5	32.7
12–24 years	831 326	36.1	46.9	12.6	3.2	0.8	0.4
25–34 years	567 328	14.1	39.8	27.6	12.0	4.3	2.1
35–44 years	594 570	29.5	49.4	15.4	4.1	1.1	0.5
45–54 years	759 189	43.7	46.2	7.9	1.5	0.4	0.2
55–64 years	697 340	46.2	45.9	6.5	1.1	0.2	0.1
65–74 years	638 067	48.7	45.4	5.2	0.6	0.1	0.0
75–84 years	362 203	50.2	44.7	4.6	0.4	0.1	0.0
≥85 years	121 016	47.0	47.1	5.4	0.5	0.1	0.0
**Region**							
Central Region	1 047 527	42.2	44.2	9.7	2.7	0.8	0.4
North Region	479 637	38.4	45.9	11.4	3.0	0.9	0.5
Zealand Region	687 298	38.8	44.7	11.6	3.3	1.0	0.5
Capital Region	1 373 937	31.4	49.6	13.6	3.8	1.1	0.4
South Region	982 642	44.2	42.5	9.4	2.6	0.8	0.5
**Municipality type**							
Peripheral	392 103	38.2	48.0	9.8	2.6	0.9	0.5
Rural	1 259 230	42.6	43.2	10.0	2.8	0.9	0.5
Intermediate	732 765	48.9	38.6	8.6	2.6	0.8	0.4
Urban	2 186 943	32.6	49.1	13.2	3.6	1.0	0.4
**Socioeconomic marker**							
Unemployed^b^	248 208	3.7	5.2	8.7	12.3	16.4	24.5
Short education^b^	497 786	13.4	10.6	6.0	3.7	3.5	5.1
Other ethnicity^b^	261 464	4.9	6.0	6.9	6.9	7.0	7.0
Single^b^	1 258 865	26.2	28.2	29.1	28.2	28.2	29.6
Children at home^b^	1 793 219	38.6	39.4	41.1	39.3	36.0	30.4
Mean income, DKK^b^	4 571 041	234 546	231 437	215 313	200 347	186 216	164 488
Income quartile 1	1 095 673	37.2	45.2	12.1	3.6	1.2	0.7
Income quartile 2	1 119 771	38.0	45.2	11.6	3.4	1.1	0.6
Income quartile 3	1 130 731	38.1	45.3	11.7	3.4	1.0	0.4
Income quartile 4	1 224 866	40.3	46.9	9.8	2.3	0.5	0.2
**Morbidity**							
CCI score, mean	4 571 041	0.276	0.246	0.165	0.120	0.110	0.132
CCI score 0	3 979 253	37.8	45.6	11.7	3.4	1.0	0.5
CCI score 1–2	496 256	42.9	46.2	8.3	1.8	0.5	0.3
CCI score 3–4	69 177	43.8	46.7	7.4	1.5	0.4	0.2
CCI score ≥5	26 355	43.7	46.5	7.7	1.4	0.4	0.3

^a^Unless otherwise specified. ^b^Percentage, mean age, and mean income calculated vertically. The number of shifts represents provider change in registrations during the analysis period. Demographic, regional, municipality, and socioeconomic markers are measured as percentages of the population in each group (*n*). The CCI score reflects the number of chronic diseases, including none. Morbidity bands (0, 1–2, 3–4, and ≥5) are derived from the CCI score. *n* in income quartiles varies due to cutoff points and data skewness. CCI = Charlson Comorbidity Index. DKK = Danish krone.

Patients from the Capital Region had the lowest COC and in most groups the highest discontinuity of care percentages, while those in intermediate municipalities had the highest COC and the lowest discontinuity of care percentages.

The percentage of individuals in each degree group who were unemployed increased with higher degrees of discontinuity of care (Table 1). Patients with the highest income (quartile 4) had the highest COC and in most groups the lowest discontinuity of care, whereas those in the lower income quartiles (1 and 2) had relatively higher percentages of greater degrees of discontinuity of care.

**Figure 2.**
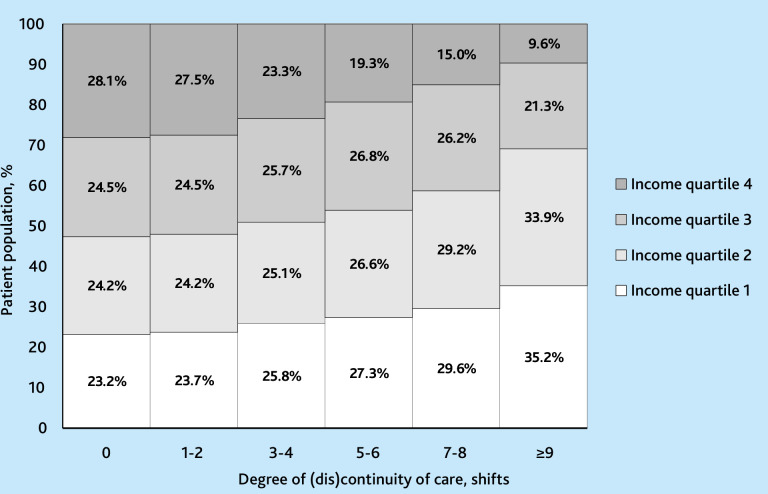
Continuity of care and varying degrees of discontinuity of care among patients in Danish general practice by patient income, 2007–2018.

Figure 2 shows the distribution of patient subgroups within each shift category as a proportion of total shifts by income.

Table 2 presents the odds of discontinuity of care levels compared with baseline COC (*n* = 1 758 753), by patient characteristic, from 2007 to 2018. Males had slightly reduced odds for lower discontinuity of care levels compared with females, but increased odds for the highest discontinuity of care levels. Younger adults (aged 25–34 years) and middle-aged adults (aged 35–44 years) had higher discontinuity of care odds compared with the reference group (aged 12–24 years), with the 25–34-year-old age group showing the highest relative odds. For both age groups, the odds of discontinuity of care increased as the number of shifts increased. Patients aged ≥45 years had lower discontinuity of care odds than the reference group, and odds declined across discontinuity of care degrees for those aged ≥65 years until the 75-84-year-old age group. The oldest age group had slightly higher odds than the second oldest at most levels, although these were still low overall.

**Table 2.** Levels of discontinuity of care in Danish general practice patients by patient characteristic, 2007–2018

	Model 1 (0 versus 1–2 shifts)		Model 2 (0 versus 3–4 shifts)		Model 3 (0 versus 5–6 shifts)		Model 4 (0 versus 7–8 shifts)		Model 5 (0 versus ≥9 shifts)		Reference
Characteristic	OR	Z	OR	Z	OR	Z	OR	Z	OR	Z	
**Demographic**											
Male	0.974^a^	–3.79	0.966^a^	−4.11	0.987	−1.17	1.022	−1.46	1.108^a^	−4.67	Female
25–34 years	1.861^a^	35.95	4.187^a^	70.13	6.675^a^	81.75	9.453^a^	79.01	11.573^a^	63.75	12–24 years
35–44 years	1.245^a^	10.73	1.363^a^	13.34	1.402^a^	13	1.682^a^	15.83	2.382^a^	17.21	12–24 years
45–54 years	0.738^a^	−19.34	0.410^a^	−41.05	0.314^a^	−43.5	0.346^a^	−29.36	0.562^a^	−10.78	12–24 years
55–64 years	0.611^a^	−25.86	0.244^a^	−51.48	0.148^a^	−58.74	0.134^a^	−48.56	0.170^a^	−29.66	12–24 years
65–74 years	0.555^a^	−24.14	0.170^a^	−50.22	0.070^a^	−60.79	0.045^a^	−55.14	0.040^a^	−41.58	12–24 years
75–84 years	0.520^a^	−20.28	0.140^a^	−45.85	0.049^a^	−51.09	0.027^a^	−41.32	0.018^a^	−30.78	12–24 years
≥85 years	0.567^a^	−16.14	0.163^a^	−41.17	0.055^a^	−44.09	0.030^a^	−28.35	0.011^a^	−17.06	12–24 years
**Region**											
Central Region	1.061	−0.58	1.03	−0.32	0.997	−0.04	0.972	−0.32	0.906	−1.06	South Region
North Region	1.121	−0.80	1.251	−1.45	1.103	−0.64	1.041	−0.26	1.042	−0.26	South Region
Zealand Region	1.363^b^	−2.61	1.836^a^	−4.79	1.906^a^	−5.05	1.841^a^	−4.82	1.795^a^	−4.32	South Region
Capital Region	1.376^b^	−2.89	1.645^a^	−4.69	1.721^a^	−5.14	1.706^a^	−5.01	1.451^a^	−3.36	South Region
**Municipality**											
Peripheral	1.027	−0.18	0.981	−0.13	1.054	−0.37	1.301	−1.79	1.928^a^	−4.35	Urban
Rural	0.829	−1.96	0.866	−1.45	0.989	−0.11	1.123	−1.19	1.492^a^	−3.97	Urban
Intermediate	0.571^a^	−5.68	0.503^a^	−7.18	0.568^a^	−5.98	0.656^a^	−4.36	0.771^c^	−2.58	Urban
**Socioeconomic status**											
Unemployed	1.137^a^	−11.95	1.471^a^	−30.40	1.791^a^	−33.72	2.026^a^	−30.5	2.534^a^	−33.11	Not unemployed
Short education	0.957^a^	−3.5	0.920^a^	−5.16	0.893^a^	−5.18	0.968	−0.95	1.303^a^	−6.13	Not short education
Other ethnicity	1.185^a^	−5.76	1.475^a^	−12.05	1.468^a^	−10.58	1.360^a^	−7.1	1.217^a^	−4.08	Not other ethnicity
Single	1.085^a^	−9.54	1.206^a^	−19.04	1.082^a^	−6.00	0.977	−1.32	0.942^b^	−2.63	Not single
Children at home	0.751^a^	−21.54	0.534^a^	−35.11	0.468^a^	−39.78	0.459^a^	−34.81	0.391^a^	−33.60	No children at home
Income quartile 1	0.959^c^	−2.15	0.987	−0.64	1.134^a^	−4.87	1.571^a^	−12.36	3.138^a^	−19.80	Income quartile 4
Income quartile 2	1.061^b^	−2.92	1.298^a^	−14.17	1.508^a^	−20.66	1.845^a^	−23.99	3.073^a^	−29.59	Income quartile 4
Income quartile 3	0.992	−0.58	1.051^a^	−3.70	1.130^a^	−8.29	1.296^a^	−12.23	1.594^a^	−14.89	Income quartile 4
**Morbidity**											
CCI score 1–2	1.046^a^	−8.84	1.113^a^	−13.23	1.150^a^	−9.68	1.171^a^	−6.41	1.400^a^	−10.44	CCI 0
CCI score 3–4	1.088^a^	−8.31	1.227^a^	−11.57	1.493^a^	−11.37	1.568^a^	−6.73	1.483^a^	−4.04	CCI 0
CCI score ≥5	1.077^a^	−5.09	1.201^a^	−6.68	1.223^a^	−3.47	1.360^b^	−2.81	1.873^a^	−4.94	CCI 0
Constant	1.605^a^	−4.84	0.458^a^	−7.94	0.107^a^	−22.54	0.0203^a^	−38.81	0.0046^a^	−48.23	
Observations, *n*	3 847 957		2 273 928		1 902 493		1 801 931		1 779 744		
Clusters, *n*	1866		1844		1832		1814		1803		
Pseudo, *R* ^2^	0.0321		0.173		0.275		0.294		0.299		
Parameters, *k*	30		30		30		30		30		
Degrees of freedom	26		26		26		26		26		
Log-likelihood	–2567713.7		−1006080.0		−369143.9		−143862.8		−79952.3		
χ^2^	2967		15573.2		28794.4		36888.6		35242.8		
*P*-value	0.000		0.000		0.000		0.000		0.000		
Akaike information criterion	5135481.5		2012213.9		738341.9		287779.7		159958.6		
Bayesian information criterion	5135836.9		2012555.1		738678.3		288114.6		160293.1		

^a^ *P*<0.001. ^b^ *P*<0.01. ^c^ *P*<0.05. z-scores represent Wald statistics (coefficient/standard error). The income was defined as the disposable income after taxes according to the applied definition. CCI = Charlson Comorbidity Index. OR = odds ratio.

Patients in Zealand Region and Capital Region were more likely to experience discontinuity of care than the reference group. Patients from intermediate municipalities were least likely to experience discontinuity of care, with odds ratios ranging from 0.571 (COC) to 0.771 (greatest degree of discontinuity of care) compared with urban residents. Peripheral and rural municipalities followed a similar trend to urban areas for lower degrees of discontinuity of care, but had a greater risk of the highest discontinuity of care (Table 2).

The results relating to socioeconomic data indicate a negative gradient in unemployment and income: patients who were unemployed and those with lower incomes had higher, and increasing, odds of experiencing greater discontinuity of care compared with the reference (income quartile 4). Patients categorised as being of a minority ethnicity had higher odds of discontinuity of care than the general population, peaking at 3–4 shifts and 5–6 shifts before returning to the initial level at the highest degree of discontinuity of care. Contrary to the authors’ hypothesis, patients with moderate morbidity (CCI score 1–2 and 3–4) had higher discontinuity of care odds than those without chronic disease (CCI score 0), with only a slight drop for CCI 3–4 in model 5 (Table 2).

## Discussion

### Summary

Whereas most previous studies have emphasised personal COC and its effects on patient outcomes,^29^ this study focused on discontinuity of care when patients are registered with a clinic, rather than an individual physician. Understanding the relative strength of association between physician- and practice-level discontinuity can inform decisions related to quality and outcome reforms,^11,29^ and highlights aspects of the quality of care delivered by a team within a general practice clinic. The present study identified demographic, geographic, socioeconomic, and morbidity-related factors associated with degrees of discontinuity of care. Discontinuity of care, measured by practice shifts, was higher among younger adults, subgroups of males, urban residents, those in Zealand Region and Capital Region, lower socioeconomic groups, and patients with higher morbidity. These findings highlight inequality in discontinuity of care among subpopulations, especially those with low income, unemployment, and chronic conditions.

### Strengths and limitations

Using longitudinal population data offers several strengths, including complete coverage, increased statistical power, generalisability/minimised sampling error, and the ability to provide longitudinal insights and detailed patient analyses.^26,30^ These strengths make the findings presented here more robust and potentially applicable to a range of general practice settings.

The large sample size allowed for the omission of traditional hypothesis testing between subpopulations of discontinuity of care and baseline COC (0 shifts), as even minor differences yielded statistically significant results.^26^ However, it is a limitation that the study’s findings are specific to the Danish general practice system, including the list system based on site registration and gatekeeping organisation.^31^ The monocentric results may not be transferable to all other countries with different general practice list systems and structures.^12,31^

Although the focus on patient characteristics provides valuable insights into those characteristics that influence discontinuity of care, it does not account for the potential impact of provider-level variables — such as provider type — on discontinuity of care . Furthermore, there is no information on the data quality. Validation of registry data is often limited by the lack of an established gold standard.^26,30^ Still, the data extracted from routine practice in the Danish regions are collected autonomously and used as the basis for patient management.^26^

As only hospital diagnoses were included in the CCI score calculation, the proportion of individuals with no recorded conditions (CCI score 0) may have been overestimated. The applied regression analysis is effective for modeling binary outcomes, providing clear interpretability through odds ratios, but it is important to be aware that logistic regression is also based on assumptions and can be sensitive to outliers.^28^ Also, it should be noted that, when a solo general practice closes, a shift is recorded more often than when a patient is reassigned a GP within a large practice.^11^

### Comparison with existing literature

The finding of lower odds for moderate degrees of discontinuity of care, and higher odds for more severe discontinuity among males, is mixed compared to previous literature, which has reported either no gender difference or greater discontinuity among both male and females.^14,32,33^ The evidence that patients aged ≥45 years had lower discontinuity of care than the reference group, align with literature showing lower discontinuity of care across age.^14,15,34^ Excluding the oldest age group (≥85 years), patients aged ≥65 years were less likely to have higher discontinuity of care than 45–64 year olds, supporting the initial hypothesis that older patients experience greater COC.^14^ This was based on evidence that older patients were found to experience significantly greater COC in general practice compared to younger age groups.^14^

Patients in rural areas have more stable care relationships, while urban patients experience more discontinuity due to circumstances such as higher physican turnover, mobility, and GP demand.^34^ Compared to the latter literature, the higher probability of discontinuity of care in Zealand Region and Capital Region, with more urbanisation and related regional health system structure, is consistent with this.^18^ Berghofer *et al* found that patients living in urban areas have a significantly higher risk of treatment discontinuity compared to those in rural areas.^18^

The low discontinuity in rural areas is consistent with the result for non-urban municipalities.^18,34^ The increased odds of discontinuity of care in peripheral and rural municipalities with the highest degree of discontinuity of care may stem from the greater prevalence of solo practitioners and unstable general practice capacity.^19^

The socioeconomic results align with most existing literature,^18,19,21,34^ although some findings remain ambiguous. Berghofer *et al* found that unemployment and low education, as proxies for socioeconomic status, were significantly associated with higher discontinuation of treatment in outpatient mental health care.^18^ Olah *et al* found reduced access to care for low socioeconomic status individuals.^21^ They interpret this as socioeconomic discrimination, suggesting that bias in appointment screening by clinic staff contributes to inequalities in COC. This structural discrimination can play a role, resulting in less attention or lower-quality care for patients of minority ethnicity.^15,21^ Other ethnicity individuals are associated with higher discontinuation, likely due to cultural or linguistic barriers.^15,18^

The increasing odds of greater degrees of discontinuity of care in lower-income quartiles (quartile 3 and quartile 2) are expected.^19–21^ It has been found that poorer communities had less access to qualified doctors and medical resources.^19–21^ In contrast, wealthier areas attract better resourced providers, resulting in a mismatch between medical need and care availability.^19,20^ Mold *et al* found that higher income and educational attainment were significantly associated with longer COC, indicating that socioeconomic advantage promotes COC, likely through better system navigation and access to transportation.^34^

Discontinuity of care has been shown to be associated with a significant increase in morbidity, particularly among those with chronic conditions, driven by reduced access to primary care and a susequent rise in hospital admissions.^3,4,7,35,36^ The findings align with the presently observed increased risk of discontinuity of care among individuals with low, moderate, and high comorbidity burdens compared to no chronic disease status, indicating that even intermediate and high morbidity levels are associated with discontinuity of care. The evidenced associations indicate that poor access to health care is linked to morbidity, as explained by the inverse care law.^19,22^ While this study assesses discontinuity of care through longitudinal changes in GP provider, most other studies use visit-based or patient-reported measures over shorter timeframes.^1,10^ The present study approach is complementary, capturing broader structural patterns in care delivery that are typically overlooked by these methods.

### Implications for practice

This study highlights the need for general practice clinicians to identify and support patients at increased risk of discontinuity — particularly those with lower income or unemployment, minority ethnic backgrounds, or chronic conditions. Although the majority of females overall had slightly higher odds for discontinuity, a small subgroup of males experienced the greatest odds for discontinuity, underscoring the importance of targeting this overlooked population. Timely recognition of these risk groups should enable interventions that strengthen patient–provider relationships and reduce negative impacts on care quality and outcomes. Notably, even those with moderate chronic illness had high levels of discontinuity, indicating that continuity strategies must extend beyond patients with complex needs. Routine chronic disease reviews are a practical opportunity to reinforce stable therapeutic relationships and ensure consistent care delivery. In addition, the increased risk for discontinuity of care in terms of many shifts in peripheral and rural municipalities likely highlights the need for targeted interventions, such as incentivising GP retention. In contrast, the lower odds of discontinuity of care in intermediate municipalities may underscore the benefits of these municipalities. Policymakers could focus on replicating conditions in the best-performing regions and municipalities to reduce discontinuity of care disparities. In contexts with frequent provider turnover — such as urban or rural areas — clinicians must adopt structured handover procedures and maintain up-to-date, shared clinical records to preserve continuity despite changes in personnel. By actively addressing these risk factors in everyday practice, GPs can foster more equitable, coordinated care and contribute to improved patient outcomes and satisfaction.

## Data

Data are protected by Statistics Denmark. Application to access these data must be made to Danish Regions and Statistics Denmark in collaboration with a Danish research institution.
